# Liver Transplantation for Autoimmune Hepatitis: Infections are the Major Cause of Mortality

**DOI:** 10.5152/tjg.2025.0201252

**Published:** 2025-03-03

**Authors:** Nilay Danış, Fulya Günşar

**Affiliations:** 1 Dokuz Eylül University Department of Internal Medicine, Division of Gastroenterology, İzmir, Türkiye; 2 Ege University Department of Internal Medicine, Division of Gastroenterology, İzmir, Türkiye

We read with great interest the article by Sağlam et al. titled “Liver Transplantation for Autoimmune Hepatitis: 20 Years of Tertiary Centre Experience,” published in your journal.^1^ As İnonu University has the busiest liver transplantation program in Türkiye, their experience is very valuable for the literature. This study reported on 51 patients who underwent transplantation due to autoimmune hepatitis between January 2002 and December 2021. Although there has been a relative decline in viral hepatitis as a cause of liver transplantation in Türkiye over recent decades, hepatitis B remains the most prevalent etiology for cirrhosis, hepatocellular carcinoma, and the need for liver transplantation.^2-5^ Given that autoimmune hepatitis is not among the most common conditions requiring liver transplantation in Türkiye, we would like to contribute our experiences and insights in this area.

This article is important because, despite the increase in publications related to autoimmune hepatitis in Türkiye over the past decade, the overall output remains insufficient.^6^ Consequently, the authorship of a study focused on contribute an activation in this area of research.

One of the most striking results of the study, biliary complications were observed 55% of tranplanted patients. Biliary complications are regarded as major problem of orthotopic liver transplantation, significantly affecting patient outcomes. Factors such as ischemia, rejection of donor tissue, and the recurrence of the underlying disease all play a role in the development of these complications. The incidence of biliary complications in LDLT ranges from 15% to 30%, this rate is significantly higher than in deceased donor liver transplantation (DDLT), which ranges from approximately 10% to 15%.^7^ Since LDLT consisted of 95% of the donors in this article, this rate of biliary complications could be the result of the high rate of living donors. In addition, the fact that the cadaveric transplantation rate is low in our country, forces the limits of donors or recipients for LDLT and can be probably caused by the high rate of biliary complications.

Sağlam et al mentioned that during a median 2.22 years follow-up, 9 of 51 patients had died after tranplantation, six of them (66%) had died due to systemic infections. That infections are the most common cause of death, is not surprising, as patients with autoimmune hepatitis are often exposed to long-term immunosuppression prior to transplantation. Consequently, the impaired recovery of the immune system post-transplant, combined with the significant stress of undergoing a major surgical procedure like liver transplantation, may contribute to this outcome.

Moreover, the persistence of the underlying autoimmune condition despite liver transplantation poses risks for acute rejection and recurrence of the underlying autoimmune hepatitis. In the study group of Sağlam et al, rejection had occurred in 11 patients (21.56%) and both rejection and recurrent autoimmune hepatitis (rAIH) had developed in 3 patients (5.88%). The rejection and rAIH rates in this study appeared to be lower than those reported in the literature.^8^ It could be related to short median follow up and the type of immunosupresive treatments. It is also important to consider that some patients who undergo transplantation due to drug-induced toxic hepatitis may, in fact, have undiagnosed autoimmune hepatitis as the underlying cause. It is well-known that one of the triggers for autoimmune hepatitis can be certain medications and dietary supplements. The precipitating factors for autoimmune hepatitis in patients considered for transplantation due to drug-related causes may stem from these medications or supplements. This possibility should be particularly emphasized during the reduction of immunosuppression aimed at minimizing the recipient’s exposure to immunosuppressive agents post-transplantation, as it may trigger latent immunity. Consequently, the immunosuppressive drug levels for patients transplanted due to autoimmune diseases may need to be maintained at a relatively higher level.

While there are several case reports suggesting an increase in autoimmune hepatitis following COVID-19 vaccination, there are also studies indicating that the incidence has not risen post-COVID-19 outbreak, which has impacted the entire globe. In fact, it is well-established that known autoimmune diseases can be triggered after viral infections.^9^ However, the evidence surrounding this issue remains inconclusive. Whether we will observe an uptick in liver transplants related to autoimmune hepatitis in the future remains to be seen; time will provide clarity on this matter.

In conclusion, while liver transplantation for autoimmune hepatitis constitutes only a small portion of liver transplants performed in Türkiye, the potential increase in cases of autoimmune hepatitis warrants further research and deeper investigation into this area.

## Data Availability

The data that support the findings of this study are available on request from the corresponding author.
